# Tracking the Impact of Age and Dimensional Shifts on Situation Model Updating During Narrative Text Comprehension

**DOI:** 10.3390/jemr18050048

**Published:** 2025-09-26

**Authors:** César Campos-Rojas, Romualdo Ibáñez-Orellana

**Affiliations:** Facultad de Filosofía y Educación, Instituto de Literatura y Ciencias del Lenguaje, Pontificia Universidad Católica de Valparaíso, Valparaíso 2370100, Chile; romualdo.ibanez@pucv.cl

**Keywords:** aging, situation model updating, eye-tracking

## Abstract

Studies on the relationship between age and situation model updating during narrative text reading have mainly used response or reading times. This study enhances previous measures (working memory, recognition probes, and comprehension) by incorporating eye-tracking techniques to compare situation model updating between young and older Chilean adults. The study included 82 participants (40 older adults and 42 young adults) who read two narrative texts under three conditions (no shift, spatial shift, and character shift) using a between-subject (age) and within-subject (dimensional change) design. The results show that, while differences in working memory capacity were observed between the groups, these differences did not impact situation model comprehension. Younger adults performed better in recognition tests regardless of updating conditions. Eye-tracking data showed increased fixation times for dimensional shifts and longer reading times in older adults, with no interaction between age and dimensional shifts.

## 1. Introduction

Imagine reading a story where two friends are chatting in a busy café. In the next sentence, the narrative shifts and suddenly places them walking together through a quiet park. Instantly and without conscious effort, your mind relocates the entire scene: the sounds, the setting, even the mood of the conversation. This seamless adjustment of the mental representation of a story illustrates what researchers call situation model updating. While this process may feel effortless, it can be affected by cognitive factors such as working memory, attention, and processing speed—all of which are known to decline with age. Research interest combining aging and language processing has resulted in a growing number of studies, many of which have paid special attention to the way age related deficits affect comprehension, or more specifically, how they affect the construction of a situation model during narrative texts comprehension [1,2,3,4]. A situation model is understood as a cognitive representation of the situation described in the text, which results from the integration of the text information and the comprehender’s background knowledge. Since the introduction of the situation model concept [5], reading comprehension theories and models have been developed to explain not only how comprehenders construct this mental representation but also how they update it during comprehension.

### 1.1. Event-Indexing Model (EIM)

According to the event-indexing model (EIM) [6,7], events are pivotal in the comprehension of narratives and are incorporated into the integrated situation model by means of five situational dimensions that comprehenders track (time, space, protagonist, causation, motivation) and use to index the incoming events. According to Zwaan and Radvansky [6], the more indexes a new event shares with the integrated model, the easier it will be integrated. The EIM also states that during comprehension, readers monitor updating. Therefore, when a discontinuation of the event because of a change in any of the five dimensions is noticed, the situation model is updated [7].

### 1.2. Event Segmentation Theory (EST)

Another influential account of situation model updating is offered by the event segmentation theory (EST) [8]. EST proposes that people naturally divide their continuous stream of experience into meaningful units, or “events.” This segmentation process is guided by predictions: as long as what happens next can be accurately anticipated, the current event model remains active. However, when ongoing activity becomes less predictable—because expectations are violated or circumstances change—a boundary is perceived that marks the end of one event and the beginning of another. At this boundary, the current event model is closed and a new one is created to represent the unfolding situation [9,10]. In this way, EST explains how comprehension is structured around event boundaries, highlighting the updating of the situation model whenever predictions about the unfolding activity fail.

### 1.3. Updating Mechanisms

Both the event-indexing model (EIM) and the event segmentation theory (EST) agree that updating a mental representation requires extra working memory resources and therefore tends to slow down reading [11]. However, they differ in how this updating takes place. According to the EIM, updating is incremental: only the dimension that has changed is revised. For instance, if a story character moves from the kitchen to the garden, the location dimension is updated while other dimensions—such as the character’s identity or goals—remain unchanged and accessible in working memory [6,7]. In contrast, EST proposes global updating: when an event boundary is detected, the reader constructs an entirely new situation model, which means that all prior information becomes less accessible and may need to be retrieved from long-term memory [2,8]. As a result, global updating is expected to produce greater processing costs than incremental updating. In this way, the two theories highlight complementary mechanisms: EIM emphasizes efficient, dimension-specific integration, while EST focuses on comprehensive restructuring of the situation model at event boundaries.

The literature has also demonstrated that situational model updating may be affected by different factors, some related to the text and others related to readers [10]. Regarding text-related factors, different studies have usually measured reading times to investigate the effect of dimensional shifts, such as spatial [12,13] or character [14] on the way the situation model is updated. While evidence generally shows that reading times increase with event shifts, this pattern has not been consistently observed [15]. For example, while some research has shown that spatial shifts result in longer reading times [16], other studies have found no significant difference [12]. Similar results have been observed for time, character, and goal dimensions. Reader-related factors have also been widely studied; they have been a central focus of cognitive aging research during the past decade [10].

### 1.4. Aging and Situation Model Updating

A primary assumption in the field is that situation model updating may vary as a function of cognitive decline associated with aging [17]. This decline in cognitive capacity has been demonstrated by studies which have observed differences between young and older adults in processing speed [18], inhibitory control [19], attention [20], and long-term memory capacity [21]. Due to its central role in language processing and particularly in the construction and updating of the situation model, special attention has been paid to working memory [22]. Additionally, it has been shown that older adults perform poorly when processing texts at the surface and textbase level [23]; and that the age effect observed at shallow levels is not such at the situation model level [1]. These findings suggest that, although domain-general declines constrain the resources available for comprehension, not all levels of text representation are equally affected.

Interested in the interaction between reader-related factors (age) and text-related factors (dimensional shifts), Bailey and Zacks [2] conducted a study looking at how young and older adults make use of incremental and global updating mechanisms when reading narrative texts that contain changes in characters and spatial locations. Self-paced reading techniques and recognition probes were used. Participants (40 older adults and 37 young adults) read narrative texts systematically controlled for changes in character (i.e., character shifts), location (i.e., spatial shifts), and for no changes (no shift). Authors predicted that older adults would rely more upon incremental mechanisms than young adults. However, the results showed that older adults took longer reading times in updating and took longer response times in answer recognition probes about information related to a dimension that did not undergo a change. These findings were interpreted as evidence that older adults are more likely to rely on global updating mechanisms, whereas younger adults tend to use incremental ones, potentially due to age-related declines in working memory. However, since the study did not assess working memory in older adults, this interpretation cannot be directly confirmed. Another unexpected finding was that readers did not significantly slow down when they encountered sentences involving character or spatial shifts, compared to those without any shifts. One possible explanation for this observation could be the limitations of reading time measures and self-paced reading techniques. In the current study, texts were presented one sentence at a time, with participants advancing to the next sentence by pressing the spacebar. This method may influence reading behavior for two main reasons. First, the inability to backtrack within the text may lead readers to adjust their reading strategies. Second, the repetitive task of pressing a button to progress through the text sentence by sentence introduces additional cognitive load, which could contribute to a decrease in reading speed [24]. These possible limitations could be overcome by using eye-tracking techniques.

### 1.5. Reading Processes and Eye Movements

One of the key advantages of eye-tracking is its capacity to capture the temporal dynamics of reading. In contrast to offline tasks that provide only post hoc outcomes, or to sentence-by-sentence self-paced reading paradigms that disrupt the natural flow of text presentation (as in Bailey & Zacks [2]), eye movements offer a continuous record of how readers allocate attention and process information as they progress through a narrative [25,26]. This temporal precision makes it possible to examine not only how quickly new information is encoded, but also how readers monitor and regulate comprehension, for example, through regressions that reveal moments of reanalysis and repair.

Different eye-tracking measures are associated with distinct stages of processing. Early measures, such as first-fixation duration—the time of the initial fixation on a word—and gaze duration—the sum of consecutive fixations before moving away—are sensitive to lexical access, word recognition, and initial integration processes [27]. Later measures capture more global aspects of comprehension: total reading time, defined as the cumulative time spent on a region including re-fixations, reflects deeper semantic and discourse-level integration, while regressions, or eye movements back to earlier text, are markers of coherence monitoring and reanalysis [28]. Beyond their descriptive value, these complementary measures provide a methodological framework for linking observable eye movements to the underlying cognitive operations involved in reading comprehension. By separating early lexical-semantic access from later coherence-building and reanalysis, eye-tracking indices clarify which aspects of processing are most affected under different textual conditions. This methodological rationale strengthens the present study, as it allows us to examine not only whether processing costs arise, but also when in the time course of reading they emerge, offering a more precise complement to traditional offline tasks or sentence by sentence methods.

Relatively few studies have used eye movements to directly examine situation model updating. Notably, Swets and Kurby [29] demonstrated that event boundaries modulate fixation times and regressions, indicating sensitivity to event structure. This suggests that eye-tracking, while well established for studying general text processing [26,27], provides a promising yet underused tool for testing theoretical predictions about updating during narrative comprehension.

## 2. Materials and Methods

### 2.1. The Present Study

An experiment with a between (older vs. young) and within-subject (no shift vs. spatial shift vs. character shift) design was carried out to test the effect of dimensional changes on situation model updating by young and older adults. This study contributes to the understanding of situation model updating by incorporating eye-tracking techniques to traditional measures (recognition probes, and comprehension questions). Besides, unlike the study by Bailey and Zacks [2], we also measured the working memory capacity of both young and older adults.

Building upon previous findings, we expected that regardless of older adults’ working memory capacity, no differences would be observed in the comprehension questions between groups [1]. This expectation is consistent with prior research showing that situation model construction tends to be preserved in older adults despite age-related declines in other cognitive resources [1,10]. Furthermore, we expected that young adults would be more accurate than older adults in the recognition probes related to unchanged dimensions, due to the use of incremental situation model updating mechanisms [2]. This prediction follows from the event-indexing model, which posits dimension-specific up-dating [6,30], and from evidence that older adults rely more on global updating as a compensatory strategy [2]. Regarding eye movement measures, we expected that both groups would have longer fixation times and more regressions in conditions with character shifts and spatial shifts in contrast with the no-shift condition. This expectation reflects the sensitivity of eye movements to situational changes in text [27,29], and supports the idea that event boundaries elicit processing costs due to model updating. Finally, it was also expected that older adults would exhibit longer fixation times and more regressions at event boundaries in comparison to young adults..

### 2.2. Participants

Forty-three young adults (36 females; age range = 18–30 years; mean age = 21.6 years; SD = 2.54 years) were recruited from Pontificia Universidad Católica de Valparaíso (PUCV). Forty older adults (26 females; age range = 60–82 years; mean age = 65.9 years; SD: 7.23 years) were recruited from two different senior activity organizations (SENAMA and Adulto Senior PUCV). The number of participants was determined with reference to Bailey and Zacks [12], who tested 37 young adults and 40 older adults in their study of situation model updating. We adopted a slightly larger sample following this precedent. Older participants were screened for dementia using the Short Blessed dementia screen (Short Blessed score < 7) [31]. All the participants were Chilean, native speakers of Spanish, fully educated, and had normal or corrected-to-normal vision. The Bioethics and Biosafety Committee at the PUCV approved the study. Each participant gave their written consent.

### 2.3. Apparatus

Eye movements were recorded monocularly in the right eye using an EyeLink Portable Duo (SR Research Ltd., Ontario, ON, Canada) at 500 Hz sampling frequency. The stimuli were presented on a 16” Notebook Gamer Rog Zephyrus M16 (Micro-Star International, New Taipei City, Taiwan), with refresh rate of 100 Hz and a resolution of 1920 × 1080 pixels. Participants were seated 70 cm from the screen, and a chin rest was used to stabilize the head.

### 2.4. Materials

#### 2.4.1. The Reading Span Task

The reading span task involves presenting participants with series of semantically unrelated sentences [32]. Participants read each sentence aloud at their own pace and then recall the final word of each sentence in the order they were presented. The task starts with series of 2 sentences and increases up to 6 sentences per series, with 3 series at each level, totaling 60 sentences of 13 to 16 words each. Sentences are shown on a computer screen for about 5 s, with each card displaying only one sentence to prevent repetition strategies and to align with working memory limits. For example, “Tired of the class’s bad behavior, the teacher went to complain to the principal” (Cansada del mal comportamiento de la clase, la profesora fue a quejarse al director). To avoid facilitating recall through associative strategies, sentences and their final words are designed to be unrelated. For instance, “The tourist quickly photographed the old building before the bus departed” (El turista fotografió rápidamente el edificio antiguo antes de que saliera el bus). The end of each series is marked by a blank screen, signaling participants to recall the words. Participants advance to the next level if they correctly recall the final words in at least two of the three series at the current level. The test concludes when they fail all three series at a level.

#### 2.4.2. The Eye-Tracking Reading Task

For the eye-tracking reading, all the stories were adopted from Bailey and Zacks [2] research and translated into Spanish by a professional translator. Further, the translations were validated by experts.

All participants were given one practice story about a visit to the aquarium and 2 of 4 experimental texts about (1) touring a castle, (2) Christmas shopping, (3) a trip to the zoo and (4) visiting a relative in the hospital (font: Courier New, font size: 14, line spacing: 2). The practice story consisted of 23 sentences (264 words), while the length of the four experimental texts varied from 90 to 92 sentences (ranging from 1277 to 1500 words). All of the experimental texts contained 12 trials, each of which was made up of a sentence containing a probe phrase, 3 filler sentences, a target sentence, a filler sentence, and a recognition probe phrase (see Appendix A). The sentences containing the probe phrase in the story were near the beginning of a new event (presented in either sentence 1, 2, 3 or 4 of a new event) and included a phrase either related to the characters in the story (e.g., “gafas gruesas”, “thick glasses”) or to the spatial locations in the story (e.g., “cerca del techo”, “near the ceiling”). The three filler sentences included information pertinent to the narrative but did not introduce significant changes across the situation models dimensions, such as characters, space, goals, objects, and time. The 12 target sentences could contain either a character shift, spatial shift, or no shift. Eye movements were measured only in the target sentences. Similarly to the original texts, the length of each target sentence varied (mean of characters per condition: no shift (M = 75.7); spatial shift (M = 97.96); character shift (M = 96.5). An additional filler sentence was added after the target sentence to avoid wrap-up effects [33]. It contained information relevant to the storyline but no major changes along the dimensions represented in situation models. The recognition probe phrases were displayed immediately following each trial on a separate screen, and they could either be the targets (e.g., “a lo largo,” “along its length”) or foils (e.g., “oficina espaciosa”, “spacious office”). Target and foil probe phrases were matched on syllable length (3 or 4 syllables in length).

We divided each story into 12–13 blocks for presentation. Block division locations were determined by the number of trials in the text.

### 2.5. Procedure and Design

Participants were tested individually and were not informed of the experiment’s purpose upon arrival, except that it involved reading with an eye tracker and a memory test. The specific objective of the task was only revealed to them after the experiment. Prior to commencing the experimentation, each participant signed an informed consent form. Following this, the eye tracker was set up, and a nine-point calibration screen was conducted for each participant. Calibration was accepted if average error was <0.50°. Participants were instructed to read the whole trial at their own pace and indicate their readiness to proceed to the next one by pressing a keyboard button. After they completed reading a full trial displayed on a single screen, they were then presented with a warning signal (#####) in the center of the screen for 500 milliseconds, followed by a probe phrase. The practice story included two probe phrases, while each of the four experimental texts contained 12 probe phrases. Among the 12 probe phrases in the experimental texts, 6 were character probe phrases, and 6 were spatial probe phrases. There were six different trial types created by combining the type of target sentence (no shift, character shift, or spatial shift) with the type of probe phrase (character probe or spatial probe). Therefore, we manipulated whether the recognition probe phrase was presented before or after the updating process. In other words, some probe phrases were presented within the same event as they were introduced, such as after a no shift (character or spatial probes after no shift). These trials likely assessed responses before the situation model updating. Other probe phrases were presented following an event boundary, such as a shift on the probed dimension (character probes after character shifts or spatial probes after spatial shifts) or a shift on the other dimension (character probes after spatial shifts or spatial shifts after character probes). Probe phrases remained on the screen until a response was recorded. Participants were instructed to press, as quickly as possible, the green button if they had read the probe phrase in the previous trial and the red one if they had not read the phrase. The responses were recorded, and there was no feedback given. Right after pressing a button, the subsequent trial in the narrative appeared on the screen. The assignment and the order of the texts and the assignment of probe phrases to the target or foil condition were counterbalanced across age groups. Therefore, within each age group (young and older), there were four groups that differed in text order (1 vs. 2) and probe phrase assignment (A vs. B), with approximately 10 participants in each group. After responding to the recognition probe, the subsequent trial was presented. Once the participants finished reading the entire story, they answered two true/false reading comprehension questions aimed at evaluation situation model construction. A Spanish version of the reading span task followed the reading task [34], and the experimental sessions typically lasted 30–40 min.

### 2.6. Data Preparation

For the Spanish adaptation of the reading span task, scoring is as follows: at levels above 2 (2-sentence series), each series is awarded 2 points if all words are recalled in the correct order, and 1 point if the order is incorrect. Since responses cannot start with the last word of the series (as specified in the instructions), unordered responses are not possible at level 2. Therefore, each correct series at this level is worth only 1 point. The scores obtained are then weighted according to their level, meaning each score is multiplied by the level number. Finally, the overall score is calculated as the sum of the partial scores from each level.

Regarding eye-tracking data, fixations shorter than 80 ms were either merged with a nearby fixation (if the distance between the fixations was <1°) or removed from the data with Data Viewer software [35]. In addition, we removed data points within the top 1% and bottom 1% of the distribution. Four different eye-tracking measures were computed for the no shift, spatial shift and character shift sentences from the eye movement data: (1) first run dwell time, all fixations made on an area of interest before abandoning it to the left or to the right [36]; (2) total fixation times, the total sum of all fixations made in an area of interest [36]; (3) regression in, checks whether a given interest area received at least one regression from later parts of the sentence (4) regression out, whether at least one regression was made from the current interest area to previous parts of the sentence prior to leaving that interest area in a forward direction. To normalize the data; the total fixation times measure was logarithmically transformed. To approach the length differences between the target sentences, following the approach of previous studies [2], a regression analysis was conducted to control for the potential effect of differences in the number of characters in target sentences across dimensional shift conditions on fixation times. Fixation times for each eye-tracking temporal measure were regressed against the number of characters of the sentence, and the residuals from these regressions were subsequently used in the linear mixed models. Due to calibration issues encountered during the eye-tracking tests, we conducted the analyses with 38 older adults and 41 younger adults. Despite these adjustments, we meticulously maintained the integrity and rigor of our data collection and analysis to ensure the reliability of our findings.

### 2.7. Data Analysis

The Mann–Whitney U test was used to analyze scores of working memory and comprehension questions. This non-parametric test was selected because it does not assume normality of the distributions and is robust to differences in variance between groups, which was particularly relevant given the observed variability in the working memory scores. The use of this test therefore provided a reliable assessment of group differences while minimizing the influence of potential violations of parametric assumptions [37]. Recognition probe data were analyzed with general linear-mixed models (GLMM). Eye-tracking data were analyzed with linear mixed-effects models (LMM) and general linear-mixed models (GLMM) using the lme4 package [38] in the RStudio statistical software (Version 4.1.1) [39]. Separate models were built for the recognition probe and each eye-tracking measure. Age (Young adults vs. Older adults) and Dimensional change (no shift vs. spatial shift vs. character shift) were fitted to each model as a deviation coded fixed effect variable. Models were also created including working memory as a fixed effect, but no statistically significant effect was observed, so it was subsequently removed. Participants and items were entered to the models as random intercepts [40]. The maximal random structure was fitted to the model [41]. When the full random structure failed to achieve convergence, the model’s random structure was systematically reduced from the top, beginning with the correlations among factors [42]. We used the Anova() function from the car package to perform Wald tests on the linear mixed-effects model. This allowed us to assess the significance of fixed effects, including main effects and interactions, while accounting for the specified model structure. Determining the precise degrees of freedom for the statistics computed by LMMs is difficult, resulting in difficulties when trying to establish precise *p*-values [40]. Therefore, degrees of freedom or *p*-values are not reported; statistical significance at the 0.05 level is indicated by values of the |t or z| > 1.96 [43]. This study’s design and its analysis were not pre-registered. The materials and the de-identified data are available in OSF. (https://osf.io/zpxbg/ accesed on 10 September 2025)

## 3. Results

In this section, we present the results in line with our initial expectations. We first examine the findings related to working memory and comprehension performance in both groups. Next, we explore the effects of age and dimensional changes, as revealed through recognition probe outcomes and eye-tracking measures.

### 3.1. Age, Working Memory and Comprehension

The comparison between young and older adults on the working memory and comprehension measures can be observed in Table 1. 

As shown in Table 1, young adults outperformed older adults in the reading span task, with significantly higher scores in working memory capacity. This difference was confirmed by the Mann–Whitney U test, which revealed a highly significant effect of age group on working memory performance (*p* < 0.001). By contrast, no significant differences were observed between groups in comprehension, suggesting that reduced working memory capacity in older adults did not translate into poorer performance on comprehension questions. These results support Hypothesis 1, showing that comprehension accuracy did not differ between young and older adults, even when older participants had lower scores on the reading span task. This suggests that situation model construction is preserved in aging despite differences in working memory capacity.

### 3.2. The Effect of Age and Dimensional Change Through Recognition Probe Measures

**Figure 1:**
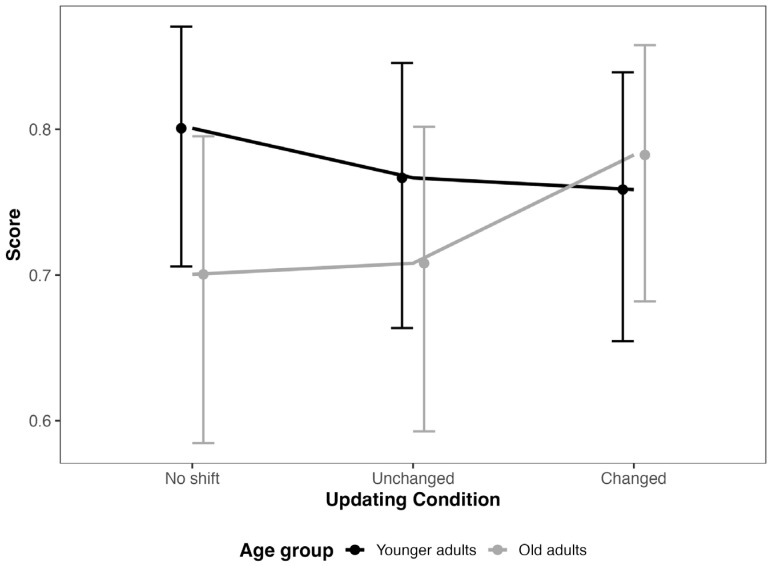
*Model estimates for total reading time in the target sentences*. Note: error bars represent 95% CI.

The results of the general linear-mixed model revealed a significant main effect of age, with older participants exhibiting lower odds of a correct response compared to younger participants (Odds Ratio = 0.58, SE = 0.13, CI [0.38, 0.90], z = −2.45). The main effect of probe type was not significant (Unchanged: Odds Ratio = 0.82, SE = 0.29, CI [0.41, 1.64], z = −0.57; Changed: Odds Ratio = 0.78, SE = 0.28, CI [0.39, 1.56], z = −0.69). However, the interaction between age and probe type was significant for the Changed probe (Odds Ratio = 1.97, SE = 0.53, CI [1.16, 3.34], z = 2.50), suggesting that the effect of age on accuracy depends on the type of probe. Wald tests confirmed these findings. There was a significant main effect of age (χ^2^ = 6.02, df = 1, *p* = 0.014) and a significant interaction between age and probe type (χ^2^ = 6.46, df = 2, *p* = 0.040). The main effect of probe type was not significant (χ^2^ = 0.54, df = 2, *p* = 0.762). These results indicate that while age influences task performance overall, the interaction with probe type is critical, particularly for Changed probes, reflecting the significant interaction effect as can be seen in Figure 1. The findings support Hypothesis 2: young adults were more accurate than older adults on probes related to unchanged dimensions, consistent with the use of incremental updating mechanisms. The lower accuracy of older adults on these probes may reflect a greater reliance on global updating, as proposed by Bailey and Zacks [2].

### 3.3. The Effect of Age and Dimensional Change Through Eye-Tracking Measures

For the sake of conciseness, we report only eye-tracking measures that exhibit statistically significant effects. For the same reason, the results of the regressions in and regressions out measures are not reported in this section.

**Figure 2:**
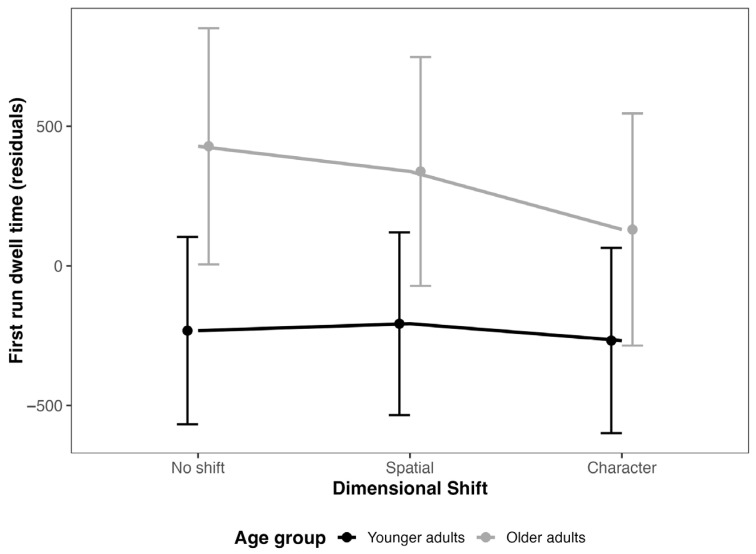
*Model estimates for first run dwell time in the target sentences*. Note: error bars represent 95% CI.

The results of the linear mixed model for the first run dwell time (see Figure 2) revealed a significant main effect for age (β = 660.50, SE = 212.81, CI [243.10, 1077.89], t = 3.10), indicating that older participants exhibited longer first run dwell time compared to younger participants. The main effect of dimension was not significant (Spatial: β = 25.00, SE = 169.42, CI [−307.29, 357.30], t = 0.15; Character: β = −35.42, SE = 171.64, CI [−372.06, 301.23], t = −0.21). Similarly, the interaction between age and dimension did not reach significance (Spatial: β = −115.25, SE = 175.37, CI [−459.20, 228.71], t = −0.66; Character: β = −262.81, SE = 177.92, CI [−611.77, 86.14], t = −1.48). Wald tests confirmed the significant main effect for age (χ^2^ = 9.63, df = 1, *p* = 0.0019), while the main effect of dimension (χ^2^ = 0.14, df = 2, *p* = 0.932) and the interaction between age and dimension (χ^2^ = 2.20, df = 2, *p* = 0.333) were not significant. These findings suggest that age independently influences first run dwell time, while dimension and its interaction with age do not significantly contribute to the model.

**Figure 3:**
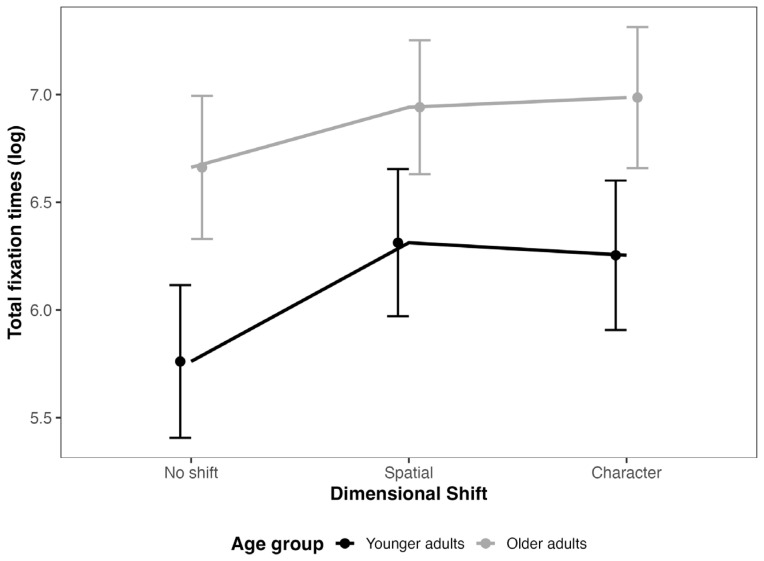
*Model estimates for total reading times in the target sentences*. Note: error bars represent 95% CI.

The results of the mixed-effects model for total fixation times (see Figure 3) revealed significant main effects for age (β = 0.90, SE = 0.23, t = 3.94) and dimension (Spatial: β = 0.55, SE = 0.19, t = 2.90; Character: β = 0.49, SE = 0.19, t = 2.57) compared to the “No shift” baseline. Older participants exhibited longer total fixation times than younger participants, and fixation times were longer in the Spatial and Character dimensions compared to the No shift condition. The interaction between age and dimension was not significant (Spatial: β = −0.27, SE = 0.21, t = −1.27; Character: β = −0.17, SE = 0.22, t = −0.77), suggesting that the effects of age were consistent across dimensions. Wald tests results confirmed significant main effects for age (χ^2^ = 15.54, df = 1, *p* < 0.001) and dimension (χ^2^ = 9.88, df = 2, *p* = 0.007), but no significant interaction between age and dimension (χ^2^ = 1.62, df = 2, *p* = 0.445). This analysis corroborates the independent contributions of age and dimension to total fixation times, with no evidence of an interaction. See Figure 3. The eye-movement results support Hypothesis 3, as both groups exhibited longer fixation times and more regressions in the character- and spatial-shift conditions compared to the no-shift condition. These patterns indicate that eye movements are sensitive markers of situation model updating during reading.

Overall, the results provide converging evidence for our hypotheses. Comprehension accuracy was preserved across age groups, consistent with Hypothesis 1; young adults showed higher accuracy on unchanged probes, supporting Hypothesis 2; and both groups exhibited eye-movement costs at situational shifts, in line with Hypothesis 3. Taken together, these findings indicate that while older adults may rely more on global updating, situation model construction remains robust across age, and eye-tracking captures the online dynamics of this process.

## 4. Discussion

This study advances the understanding of situation model updating by complementing previous measures (recognition probes) with eye-tracking techniques to explore how dimensional changes affect both young and older adults’ performance. Additionally, and based on the relevance assigned to the phenomenon in the literature, working memory capacity was assessed to verify its relation with any potential age effects.

Regarding general results, young adults exhibited higher levels of verbal working memory capacity compared to their older counterparts. These results are consistent with our expectations and with the extensive body of research demonstrating age-related changes in working memory capacity [44]. Older adults often struggle with tasks requiring the manipulation and retention of information, which can significantly affect their performance on working memory tests [45].

While our findings reveal significant age-related differences in working memory performance, with younger adults outperforming older adults, no such differences were observed in comprehension test results. As we expected, performance on reading comprehension questions assessing situation model construction was similar across age groups. Our results were consistent with previous studies, which demonstrate that the age effect observed at surface code and textbase performance is not such at the situation model level [1]. Our results are also in line with studies that have demonstrated that older adults are able to comprehend short and long texts [46].

In the recognition probe, we also expected younger adults to exhibit superior performance compared to older adults. These expectations were based on the findings of Bailey and Zacks [2] and were corroborated by our results. In their study, Bailey and Zacks [2] also predicted that younger adults would perform equal for No shift and Unchanged dimension and less accurate for Changed dimension, while older adults would perform more accurate for No shift condition in comparison with Unchanged dimension and Changed dimension, assuming that older adults would rely on global updating while young adults on incremental. Contrary to their expectations, they found no difference across Updating conditions. In a completely different pattern, in our results, it was observed that while younger adults exhibit no statistical differences across conditions, older adults perform significantly better in Changed condition than in Unchanged and No Shift. Our results suggest that older adults may allocate greater attention to aspects of the text associated with dimensional changes. This aligns with the idea that older adults adopt more strategic processing when confronted with such changes, potentially as a compensatory mechanism to manage the cognitive demands involved [10]. By focusing more deliberately on relevant information, older adults might better integrate and recall the changing aspects of the narrative. This idea is supported by research indicating that older adults employ strategic approaches to mitigate cognitive decline [10]. These strategies might include enhanced monitoring of text changes or increased effort to maintain situational context amidst shifting dimensions. Such an approach suggests that older adults may use their accumulated knowledge and experience to prioritize and organize information more effectively, facilitating better retrieval and comprehension of changing elements in the narrative.

Regarding eye-tracking measures, our analysis of first run dwell time revealed significant differences between young and older adults, but no notable variations between dimensional conditions. Specifically, young adults exhibited shorter first run dwell times compared to older adults, reflecting a more efficient initial processing of information. This finding aligns with previous research indicating that young individuals typically have faster and more efficient visual processing capabilities [47]. The observed delay in first run dwell times for older adults is consistent with the literature on age-related slowing of cognitive and perceptual processes [48,49]. Interestingly, the lack of significant differences between dimensional conditions suggests that the efficiency of initial processing was unaffected by the dimensional shifts. This may indicate that the main influence on first run dwell time was related to the general processing speed differences between age groups rather than specific changes in dimensional shift conditions. It also suggests that while age impacts initial processing efficiency, the impact of dimensional shifts on early visual attention might be minimal or uniform across both age groups. This finding contributes to the broader understanding of how visual attention processes interact with cognitive aging and highlights the need to consider age-related factors when evaluating initial information processing in various contexts.

Regarding total fixation times, we anticipated that older adults would demonstrate slower total fixation times compared to younger adults, in line with established findings on age-related declines in reading speed and cognitive processing efficiency [48,50]. Additionally, we expected that fixation times would increase for sentences containing character and spatial changes, with these effects potentially being more pronounced for older adults due to the additional cognitive load imposed by updating situation models [8].

Our results corroborate the expectation that older adults would exhibit slower total fixation times than younger adults, consistent with research documenting age-related reductions in processing capacity and efficiency [50]. This slower fixation times among older adults is reflective of broader age-related cognitive declines, particularly in the realm of working memory and attentional control. Moreover, our findings reveal that sentences with character and spatial changes elicit increased fixation times across both age groups. This outcome supports the notion that dimensional shifts in the narrative, whether involving characters or spatial elements, impose additional cognitive demands that necessitate longer processing times [8].

Interestingly, contrary to our initial hypothesis, the effect of these dimensional changes did not interact significantly with age. This lack of interaction suggests that while older adults exhibit slower overall total fixation times, the impact of dimensional changes on processing is similar across age groups [50]. In other words, both younger and older adults experience similar challenges when processing and integrating new information related to character and spatial shifts in the narrative. This finding indicates that the cognitive load associated with updating situation models due to dimensional changes does not vary markedly between age groups, although older adults may be generally slower in their fixations. This outcome highlights that while age affects the processing, the fundamental processes involved in updating situation models in response to narrative changes remain consistent across different age groups.

In our study, we anticipated that regressions would increase in response to dimensional shifts, given previous findings by Swets and Kurby showing that readers often use regressions as a strategy to reprocess information when integrating event changes. From this perspective, regressions are assumed to reflect additional cognitive effort to ensure coherence, particularly when updating the situation model at event boundaries. Contrary to these expectations, however, we did not observe significant differences in regression patterns either across dimensional conditions or between age groups (see Appendix E and Appendix F). This result suggests that, although regressions can serve as an index of processing difficulty in narrative comprehension, they are not a necessary marker of situation model updating. Instead, our findings support the idea that readers may rely on forward processing and incremental adaptation of the mental representation without systematically resorting to rereading.

The present findings also have theoretical implications for models of situation model updating. The longer reading times at character and spatial shifts are consistent with the event-indexing model, which posits dimension-specific incremental updating [6]. At the same time, the broader costs observed in older adults, particularly in recognition probes related to unchanged dimensions, align with event segmentation theory, which emphasizes global updating at event boundaries [8]. Taken together, these results suggest that incremental and global updating mechanisms are not mutually exclusive, but may operate in parallel, with aging modulating their relative weight.

Beyond their theoretical contribution, the present findings also have practical and methodological implications. Methodologically, the study demonstrates the value of combining eye-tracking with traditional measures (e.g., recognition probes and comprehension questions) to capture both online and offline aspects of situation model updating. This multimethod approach can serve as a template for future research on discourse processing, particularly when investigating age-related differences. Practically, understanding how older adults update situation models has implications for educational and clinical contexts, as it may inform interventions aimed at supporting reading comprehension in aging populations. More broadly, our results highlight the importance of considering both incremental and global updating strategies in designing materials or tasks that facilitate comprehension across the lifespan.

### Limitations and Future Directions

A potential limitation of the present study concerns sentence length. Although the materials were carefully designed to control the number of dimensional shifts per trial, complete equivalence in sentence length across conditions could not be achieved. To address this issue, fixation times for each eye-tracking temporal measure were regressed against the number of characters of the sentence, and the resulting residuals were subsequently entered into the linear mixed models [2]. This procedure allowed us to control for the influence of sentence length on reading times while preserving the effects of the experimental manipulations. Nevertheless, differences in sentence length remain a design constraint, and future research would benefit from achieving greater balance across conditions to further strengthen the robustness of the findings.

## 5. Conclusions

This study enhances our understanding of situation model updating by integrating eye-tracking technology with traditional recognition probes to investigate how dimensional changes impact reading performance across age groups. Our findings corroborate established research indicating age-related declines in working memory capacity, with younger adults demonstrating superior performance in tasks requiring verbal working memory. However, these differences in working memory capacity did not translate into significant variations in the comprehension of situation models across age groups.

Our results reveal that both young and older adults exhibit similar performance when engaging with dimensional changes in narrative texts, challenging the notion that age-related declines in working memory result in increased difficulties with situation model construction. The eye-tracking data further illustrate that although older adults experience slower initial processing and longer total fixation times, the impact of dimensional changes on fixation times is consistent across age groups.

Contrary to expectations, we did not find significant age-related differences in the number of regressions. These results are interesting as they may indicate that older adults employ compensatory strategies that mitigate the need for frequent revisits of previously read material. This indicates that while age affects processing speed, the fundamental cognitive processes involved in updating situation models in response to narrative changes remain robust and similar across different age groups.

Overall, these findings enhance our understanding of age-related differences in reading and narrative comprehension by highlighting that while cognitive aging affects processing speed, it does not fundamentally alter the mechanisms of situation model updating. Our study contributes to the field not only through the use of eye-tracking techniques but also by including data from less studied populations. Both the EIM and the EST, as well as their influencing factors, have predominantly relied on data from populations in the Global North. This limited perspective may constrain the validity and applicability of the findings, as they predominantly reflect WEIRD (Western, Educated, Industrialized, Rich, and Democratic) societies. Consequently, these models may be less relevant in diverse contexts, particularly in the Global South. Future research should continue exploring age-related factors with a focus on the adaptive strategies employed by older adults and by including a broader range of populations to enhance the generalizability of the results.

## Figures and Tables

**Table 1:** Working memory and comprehension performance per age group, * is an important identifier of data.

	Working Memory			Comprehension		
Group	Mean	SD	*p*	Mean	SD	*p*
Older adults	11.5	5.11	<2.2 × 10^−16^ *	0.72	0.45	<0.69
Young adults	21.9	9.75		0.74	0.44	

## Data Availability

Data are contained within this article.

## Appendix A. Example of a Trial Structure in English and Spanish

**Table:**

English Original Text	Spanish Version	Type of Sentence
Haley was especially tired of the armory, where they had been standing for nearly fifteen minutes.	Carla estaba especialmente aburrida de la armería, donde estuvieron de pie cerca de quince minutos.	Filler sentence A
However, she did have to admit that the sheer number of weapons on display in the room was impressive.	Sin embargo, sí tuvo que admitir que el número de armas puestas en exhibición en la habitación era simplemente impresionante.	Filler sentence B
The wall they were standing next to had various weapons hanging all **along its length**.	La pared junto a la que estaban parados tenía una gran cantidad de armas, que colgaban **a lo largo** de ella.	Sentence with probe phrase
Haley thought the swords looked really intimidating, and hoped they were securely fastened to the wall.	Carla pensó que las espadas se veían muy intimidantes y esperó que estuviesen apropiadamente aseguradas a la pared.	Filler sentence 1
She was sure the tour guide was explaining all the historical details, but she had stopped listening to him.	Estaba segura de que el guía les estaba explicando todos los detalles históricos, pero había dejado de escucharlo.	Filler sentence 2
She hoped he wasn’t going to talk continuously during the entire tour.	Carla hubiese preferido que el guía no hablara sin parar durante el tour.	Filler sentence 3
The group walked out of the armory and into the castle’s outdoor courtyard.	Carla salió de la armería y caminó hacia el patio exterior del castillo.	**Event boundary.** Target sentence with spatial shift
It had many trees, bushes and benches for resting.	Este contaba con muchos árboles, arbustos y bancas para descansar.	Filler sentence added to avoid wrap up effects
ALONG ITS LENGTH, ALONG THE HALL [FOIL]	A LO LARGO, A LO ANCHO [FOIL]	Recognition probe

## Appendix B. General Linear Mixed Model for Recognition Probe Accuracy

**Table:**

*Predictors*	Accuracy			
	*Odds Ratios*	*Std. Error*	*CI*	*Statistic*
(Intercept)	4.02	1.06	2.39–6.74	5.27
age [Old]	0.58	0.13	0.38–0.90	−2.45
probe [Unchanged]	0.82	0.29	0.41–1.64	−0.57
probe [Changed]	0.78	0.28	0.39–1.56	−0.69
age [Old] × probe [Unchanged]	1.27	0.34	0.75–2.15	0.88
age [Old] × probe [Changed]	1.97	0.53	1.16–3.34	2.50
Random Effects				
σ^2^	3.29			
τ_00 participant_	0.24			
τ_00 item_	0.70			
ICC	0.22			
N _participant_	83			
N _item_	48			
Observations	1945			
Marginal R^2^/Conditional R^2^	0.009/0.230			

## Appendix C. Linear Mixed Model for First Run Dwell Time

**Table:**

*Predictors*	First Run Dwell Time			
	*Estimates*	*Std. Error*	*CI*	*Statistic*
(Intercept)	−232.08	171.00	−567.46–103.31	−1.36
age [Old]	660.50	212.81	243.10–1077.89	3.10
dimension [Spatial]	25.00	169.42	−307.29–357.30	0.15
dimension [Character]	−35.42	171.64	−372.06–301.23	−0.21
age [Old] × dimension [Spatial]	−115.25	175.37	−459.20–228.71	−0.66
age [Old] × dimension [Character]	−262.81	177.92	−611.77–86.14	−1.48
Random Effects				
σ^2^	1,761,838.76			
τ_00 participant_	581,628.86			
τ_00 item_	150,276.23			
τ_11 item_._ageOld_	58,229.68			
ρ_01 item_	1.00			
ICC	0.32			
N _participant_	79			
N _item_	48			
Observations	1761			
Marginal R^2^/Conditional R^2^	0.030/0.344			

## Appendix D. Linear Mixed Model for Total Fixation Times

**Table:**

*Predictors*	Total Fixation Times			
	*Estimates*	*Std. Error*	*CI*	*Statistic*
(Intercept)	5.76	0.18	5.41–6.12	31.94
age [Old]	0.90	0.23	0.45–1.35	3.94
dimension [Spatial]	0.55	0.19	0.18–0.92	2.90
dimension [Character]	0.49	0.19	0.12–0.87	2.57
age [Old] × dimension [Spatial]	−0.27	0.21	−0.69–0.15	−1.27
age [Old] × dimension [Character]	−0.17	0.22	−0.60–0.26	−0.77
Random Effects				
σ^2^	1.24			
τ_00 participant_	0.43			
τ_00 item_	0.06			
ICC	0.28			
N _participant_	73			
N _item_	47			
Observations	712			
Marginal R^2^/Conditional R^2^	0.090/0.348			

## Appendix E. Generalized Linear Mixed Model for Regressions in

**Table:**

*Predictors*	Regressions In			
	*Odds Ratios*	*Std. Error*	*CI*	*Statistic*
(Intercept)	0.14	0.04	0.09–0.23	−7.96
age [Old]	1.11	0.30	0.66–1.90	0.40
dimension [Spatial]	1.73	0.51	0.97–3.10	1.85
dimension [Character]	1.50	0.46	0.83–2.73	1.33
age [Old] × dimension [Spatial]	0.76	0.24	0.41–1.39	−0.89
age [Old] × dimension [Character]	0.94	0.30	0.51–1.76	−0.19
Random Effects				
σ^2^	3.29			
τ_00 participant_	0.35			
τ_00 item_	0.35			
ICC	0.17			
N _participant_	79			
N _item_	48			
Observations	1797			
Marginal R^2^/Conditional R^2^	0.010/0.183			

## Appendix F. Generalized Linear Mixed Model for Regressions out

**Table:**

*Predictors*	Regressions Out			
	*Odds Ratios*	*Std. Error*	*CI*	*Statistic*
(Intercept)	0.13	0.04	0.07–0.24	−6.59
age [Old]	0.84	0.27	0.45–1.59	−0.53
dimension [Spatial]	1.31	0.48	0.64–2.70	0.74
dimension [Character]	0.81	0.31	0.39–1.71	−0.55
age [Old] × dimension [Spatial]	1.60	0.52	0.84–3.04	1.44
age [Old] × dimension [Character]	1.60	0.55	0.82–3.14	1.38
Random Effects				
σ^2^	3.29			
τ_00 participant_	0.86			
τ_00 item_	0.69			
ICC	0.32			
N _participant_	79			
N _item_	48			
Observations	1797			
Marginal R^2^/Conditional R^2^	0.014/0.331			
